# Expression of *Aspergillus niger* CAZymes is determined by compositional changes in wheat straw generated by hydrothermal or ionic liquid pretreatments

**DOI:** 10.1186/s13068-017-0700-9

**Published:** 2017-02-07

**Authors:** Paul Daly, Jolanda M. van Munster, Martin J. Blythe, Roger Ibbett, Matt Kokolski, Sanyasi Gaddipati, Erika Lindquist, Vasanth R. Singan, Kerrie W. Barry, Anna Lipzen, Chew Yee Ngan, Christopher J. Petzold, Leanne Jade G. Chan, Steven T. Pullan, Stéphane Delmas, Paul R. Waldron, Igor V. Grigoriev, Gregory A. Tucker, Blake A. Simmons, David B. Archer

**Affiliations:** 1 School of Life Sciences, University of Nottingham, University Park, Nottingham, NG7 2RD UK; 2 Deep Seq, Faculty of Medicine and Health Sciences, Queen’s Medical Centre, University of Nottingham, Nottingham, NG7 2UH UK; 3 School of Biosciences, University of Nottingham, Sutton Bonington Campus, Loughborough, LE12 5RD UK; 4 US Department of Energy Joint Genome Institute, Walnut Creek, CA 94598 USA; 5 Joint BioEnergy Institute, Emeryville, CA 94608 USA; 6 Fungal Physiology, CBS-KNAW Fungal Biodiversity Centre, Utrecht University, Uppsalalaan 8, 3584 CT, Utrecht, The Netherlands; 7 Chemical Biology, Manchester Institute for Biotechnology, University of Manchester, 131 Princess Street, Manchester, M1 7DN UK; 8 TB Programme, Microbiology Services, Public Health England, Salisbury, UK; 9 UPMC, Univ. Paris 06, CNRS UMR7238, Sorbonne Universités, 15 rue de l’Ecole de Médecine, 75270 Paris, France

**Keywords:** *Aspergillus niger*, Lignocellulose, Ionic liquid and hydrothermal pretreatments, Straw, Transcriptomic responses, CAZy, Hemicellulose, RNA-seq, Targeted proteomics

## Abstract

**Background:**

The capacity of fungi, such as *Aspergillus niger,* to degrade lignocellulose is harnessed in biotechnology to generate biofuels and high-value compounds from renewable feedstocks. Most feedstocks are currently pretreated to increase enzymatic digestibility: improving our understanding of the transcriptomic responses of fungi to pretreated lignocellulosic substrates could help to improve the mix of activities and reduce the production costs of commercial lignocellulose saccharifying cocktails.

**Results:**

We investigated the responses of *A. niger* to untreated, ionic liquid and hydrothermally pretreated wheat straw over a 5-day time course using RNA-seq and targeted proteomics. The ionic liquid pretreatment altered the cellulose crystallinity while retaining more of the hemicellulosic sugars than the hydrothermal pretreatment. Ionic liquid pretreatment of straw led to a dynamic induction and repression of genes, which was correlated with the higher levels of pentose sugars saccharified from the ionic liquid-pretreated straw. Hydrothermal pretreatment of straw led to reduced levels of transcripts of genes encoding carbohydrate-active enzymes as well as the derived proteins and enzyme activities. Both pretreatments abolished the expression of a large set of genes encoding pectinolytic enzymes. These reduced levels could be explained by the removal of parts of the lignocellulose by the hydrothermal pretreatment. The time course also facilitated identification of temporally limited gene induction patterns.

**Conclusions:**

The presented transcriptomic and biochemical datasets demonstrate that pretreatments caused modifications of the lignocellulose, to both specific structural features as well as the organisation of the overall lignocellulosic structure, that determined *A. niger* transcript levels. The experimental setup allowed reliable detection of substrate-specific gene expression patterns as well as hitherto non-expressed genes. Our data suggest beneficial effects of using untreated and IL-pretreated straw, but not HT-pretreated straw, as feedstock for CAZyme production.

**Electronic supplementary material:**

The online version of this article (doi:10.1186/s13068-017-0700-9) contains supplementary material, which is available to authorized users.

## Background

Lignocellulose, which is composed of polysaccharides and lignin, is an abundant plant raw material that is used to make liquid biofuels [1]. The polysaccharides are a source of simple sugars that can be released enzymatically by saccharification for subsequent fermentation to biofuels [2]. One barrier to cost-efficient production of biofuels is the inefficient saccharification step, which requires excessive amounts of costly enzymes produced by industry using fungi [3]. As part of an approach to improve efficiency, lignocellulosic substrates are routinely pretreated to render the lignocellulose more amenable to saccharification by enzymes [4]. Production of more efficient and cheaper enzyme mixtures would be aided by improved knowledge gained from studying the responses of nature’s lignocellulose-degrading fungi to pretreated lignocellulosic feedstocks.

Pretreated lignocelluloses offer the opportunity to study how fungi respond and adapt to major changes in the composition and structure of lignocellulose. Pretreatments consist of a physical and/or chemical process that has the aim of opening up the structure of the lignocellulose to improve the efficiency of saccharification [5]. Hydrothermal pretreatments, which are commonly used, tend to remove some hemicelluloses from the lignocellulose as well as break linkages between the polysaccharides and lignin [6]. The use of ionic liquids is an emerging pretreatment technology which facilitates the solubilisation of lignocellulose and, crucially, regenerates insoluble but now highly saccharifiable polymers [7]. In our study we pretreated wheat straw, which is a major agricultural waste residue with potential for use to produce bioethanol [8].

Previously, various untreated lignocelluloses as well as individual polysaccharides have been used to study the transcriptional response of the ascomycetes *Aspergillus niger* [9–13], *Aspergillus nidulans* [14], *Trichoderma reesei* [15–18], *Neurospora crassa* [14, 19, 20] and *Myceliophthora thermophila* [21]. Transcriptional responses of mixed cultures of ascomycete fungi with untreated lignocellulose have also been studied [22]. A limited number of studies have used pretreated substrates, namely where *A. niger* was exposed to steam-exploded bagasse [10] or where *T. reesei* was exposed to steam-exploded bagasse, wheat straw or spruce [15]. The most prominent feature of these studies was the induction of many genes encoding **C**arbohydrate-**A**ctive en**Zy**mes (CAZy, as found in the CAZy database, http://www.cazy.org/ [23]) that saccharify lignocellulose (for reviews, see Daly et al. [24] and Glass et al. [25]).

There are several major gaps in our understanding of the fungal response to pretreated lignocellulose that previous studies could not adequately address. Firstly, as fungi were exposed only to steam explosion-based pretreatment [10, 15], the effect of the majority of pretreatment-induced changes in lignocellulose composition and structure is so far unknown. Secondly, previous studies had limited temporal resolution making it difficult to delineate the temporal aspects of the response to the substrates. Kolbusz et al. [21] coined the phrase ‘cryptic CAZy’ to refer to those genes encoding biomass-degrading CAZymes that have not been shown to be induced in response to lignocelluloses. Their expression may just be temporally limited rather than being irrelevant to lignocellulose degradation. Thirdly, concentrations of sugars present in cultures with recalcitrant untreated feedstocks can be relatively low, such as in Delmas et al. [9]. Highly saccharifiable, pretreated substrates facilitate the study of the response to a different range of concentrations of those same sugars. Furthermore, the sensing of ratios of sugar concentrations rather than just concentrations of individual sugars is a recently established paradigm for fungi [26] and a broad time course facilitates exposure to many more different ratios of these sugars.

*A. niger*, a saprobic ascomycete filamentous fungus, is both a model organism and used industrially for the production of organic acids and enzymes [27, 28]. *A. niger* possesses a large repertoire of CAZymes that are active towards plant polysaccharides [13, 27]. *A. niger* has regulatory mechanisms mediated by transcription factors such as XlnR and AraR that allow the fungus to respond to the presence of polysaccharides in its environment [29, 30]. Both broad spectrum and specific inducers mediate this regulation. For example, xylose not only induces genes encoding xylanolytic enzymes but also endoglucanases via XlnR [29], whereas specific aromatic compounds induce the gene encoding the feruloyl esterase FaeB [31], which generally functions to cleave linkages that involve the inducing aromatics. Previously, we have shown a role for carbon starvation in the induction of CAZy genes during the early response of *A. niger* to wheat straw [9, 11].

In this study, we investigated the temporal responses of *A. niger* to untreated and pretreated wheat straw by measuring genome-wide transcript abundance, specific proteins and secreted enzyme activities. To our knowledge, this is the first study providing an extensive time course of the fungal responses to untreated and pretreated lignocelluloses, as well as the first in reporting the fungal response to ionic liquid-pretreated lignocellulose. We identify uncharacterised CAZy genes, regulators and transporters relevant to lignocellulose degradation by relating gene expression with exposure to the untreated and pretreated straw.

## Results

### Pretreatments changed the composition and properties of the lignocellulosic substrates

**Fig. 1:**
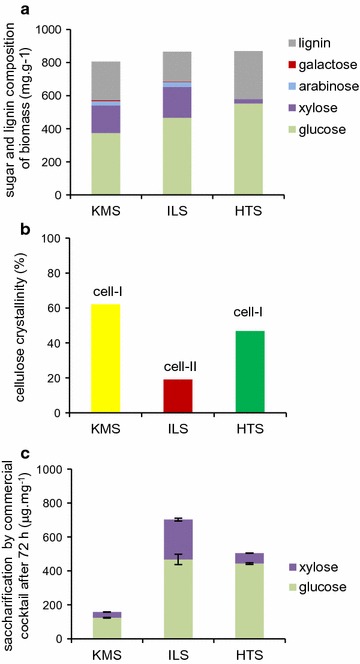
Pretreatments changed the composition and properties of the lignocellulosic substrates. **a** Substrate composition as measured from the monosaccharide profile of the acid hydrolysates of the untreated and pretreated straw, and acetyl bromide determination of lignin. **b** Crystallinity of the untreated and pretreated straw. Cell-I or cell-II refers to the predominant type of cellulose in the untreated and pretreated straw. **c** Saccharification of substrates in standard type saccharification assays as measured by release or glucose and xylose. The *error bars* represent standard errors (*n* = 3)

We generated substrates for the cultivation of *A. niger* by applying ionic liquid (IL) and hydrothermal (HT) pretreatments to knife-milled wheat straw (KMS), resulting in the production of IL-pretreated straw (ILS) and HT-pretreated straw (HTS). The pretreatments altered the composition of the straw in different ways (Fig. 1). After HT pretreatment, the amount of hemicellulose and pectin components was reduced compared to the untreated (KMS) and IL-pretreated straw (ILS), as shown by a reduction of at least tenfold in the amount of arabinose and galactose, and sixfold in the amount of xylose, as quantified in the acid hydrolysates of the straw (Fig. 1a). Both pretreatments resulted in lignin removal compared to the untreated straw when factoring in mass loss due to pretreatment: from 26 to 16% for IL and to 23% for HT. The cumulative changes in straw due to pretreatment resulted in an enrichment in lignin content in the remaining solids of HTS (290 mg g^−1^) compared to KMS (234 mg g^−1^), whereas the lignin content was depleted in the remaining solids of ILS (180 mg g^−1^) (Fig. 1a). The IL pretreatment led to a major reduction in the crystallinity of the cellulose as measured by X-ray diffraction and the regenerated cellulose recrystallized as Cellulose-II (Cell-II) in contrast to the native Cellulose-I (Cell-I) structure predominantly found in the KMS and HTS (Fig. 1b). Saccharification assays using a standard commercial enzyme cocktail confirmed that both the pretreatments improved the saccharification efficiency compared to the KMS (Fig. 1c). From both pretreated substrates, there was ~fourfold increase in glucose release per amount of solids. The increase in xylose release from the ILS (eightfold) was higher than from the HTS (twofold), reflecting the lower amounts as well as higher recalcitrance of the remaining xylan in the HTS.

### Time courses of *A. niger* responses when exposed to complex substrates

**Fig. 2:**
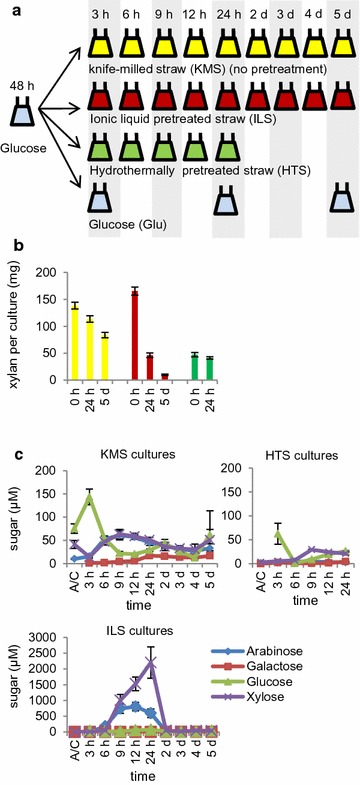
Experimental design and in-culture substrate saccharification. **a** *A. niger* was cultured with the indicated untreated and pretreated substrates for the times indicated (*n* = 3). For the cultures with the HT-pretreated straw, it was not possible to obtain sufficient RNA for RNA-seq from the cultures from the later time points. **b** Measurements of xylan in the initial substrates and residual xylan from the solids recovered from the fungal cultures after 24 h and 5 d (where available). Data are given as the mean (*n* = 3) of biological replicate flasks with *error bars* representing standard errors, or for the initial substrates as the mean with standard errors (*n* = 2) of technical hydrolysis replicates. **c** Monosaccharide profiles in the fungal culture filtrates over time. The *error bars* represent standard errors (*n* = 3). AC only = autoclaved substrate only

Pretreated and untreated straw substrates were used in shake-flask cultures to identify the fungal responses upon exposure to the substrates. Cultures of *A. niger* from glucose-rich media were washed and transferred to untreated and pretreated straw substrates as well as glucose control cultures and incubated for the times illustrated in Fig. 2a. The final time points for which cultures were sampled were dependent on whether RNA of sufficient quantity and quality could be extracted. In the cases of the HTS cultures, it was not possible to obtain RNA of sufficient quantity after 24 h. The KMS and ILS cultivations continued up to 5 days. In total, there were 27 conditions of *A. niger* cultured with a particular substrate for a certain length of time. The pH of the cultures changed over time, decreasing in the first 24 h (HTS) or 2 d (KMS and ILS) and slowly increasing after that (Additional file 1: Figure S1).

### High ILS digestibility and pentose sugar accumulation

Cultivation of *A. niger* on the untreated and pretreated substrates resulted in partial digestion of the wheat straw (Fig. 2b). The residual xylan in the solids recovered from the cultures with KMS was reduced to 82% of the initial xylan after 1 day of cultivation, and to 60% after 5 days. The rate and extent of ILS saccharification were increased in the cultures compared to KMS, as indicated by the detection of lower amounts of residual xylan in the recovered solids. For example, while the amounts of xylan initially present in the ILS and KMS were similar at ~150 mg xylan per flask, the residual xylan measured in the ILS cultures from 5 d was 10 mg per flask (6% of the initial xylan), which was eightfold less than the 83 mg measured in the KMS cultures from 5 d. This increased fungal saccharification of ILS was in contrast with HTS, which was not saccharified more than KMS, with 86% of the initial xylan remaining in the substrate after 1 day.

Increased saccharification of ILS in the cultures was supported by the concentrations of arabinose, galactose, glucose and xylose measured in the fungal culture filtrates (Fig. 2c). The concentration of the monosaccharides was generally low, at less than 100 μM. However, there was a dramatic increase in the concentrations of arabinose and xylose, to ~1–2.5 mM, in the ILS cultures at the 9-h, 12-h and 24-h time points. This accumulation confirmed that the ILS was indeed saccharified more by *A. niger* than the HTS or KMS, releasing xylose up to levels that have been reported to be repressive for CAZy gene expression [32]. These high monosaccharide concentrations affected gene expression in the fungus.

### CAZyme-encoding transcripts have highest abundance at the middle time points

**Fig. 3:**
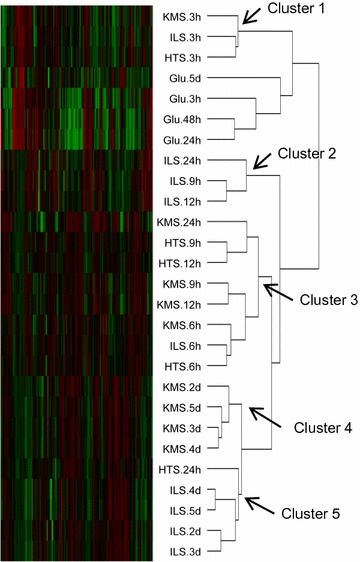
Heatmap of hierarchical clustering of CAZy transcript patterns by condition. The conditions were clustered using the log-transformed and quartile-normalised mean FPKM values of CAZy genes, excluding genes whose expression was not ≥1 FPKM in any of the time points on any media. The clusters 1–5 are referred to in the main text. A heatmap of the clustering of the conditions using the signal peptide annotated gene FPKM values can be found in Additional file 2: Table S1

In an initial analysis to provide an overview of the trends, hierarchical clustering was performed using the RNA sequencing-derived transcript levels of the CAZy genes in each of the conditions. This showed that time-dependent clustering was prominent for early (3–6 h) time points, while pretreatment-dependent clustering was prominent in middle (9–24 h) and later (2–5 days) time points (Fig. 3). The 3-h time points from untreated and pretreated straw clustered together (cluster 1). Middle time points from the ILS cultures (ILS from 9 h, 12 and 24 h) clustered together in cluster 2, separate from most of the corresponding time points from KMS and HTS cultures in cluster 3. The latter (2–5 days) time points from KMS and ILS cultures were found clustered separately (cluster 4 and 5).

**Fig. 4:**
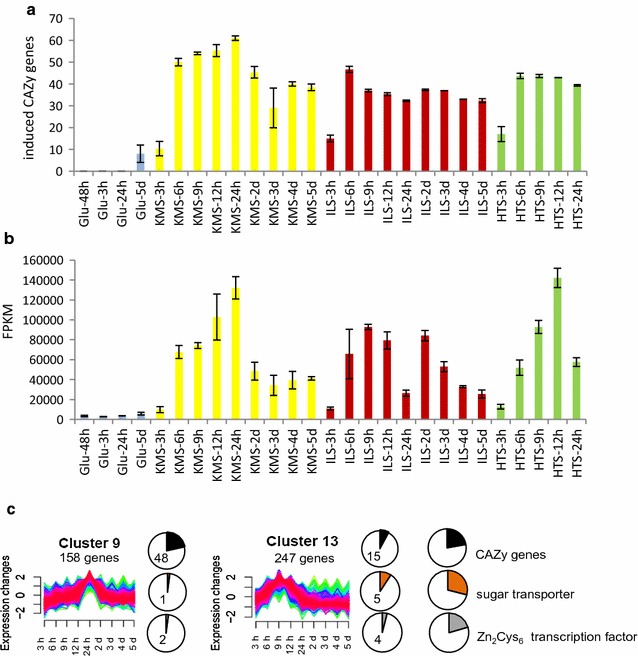
Overview of CAZy gene transcripts. **a** The number of CAZy genes that encode plant-polysaccharide-active CAZymes and is significantly induced in cultures with untreated and pretreated straw compared to the Glu 48 h control (with a DESeq *p*_*adj*_ < 0.05, FPKM of ≥50 on lignocellulose and log2 FC of ≥3).* Error bars* represent standard errors (*n* = 3). **b** The proportion of transcripts from CAZy genes that encode plant-polysaccharide-active CAZymes is expressed as FPKM value.* Error bars* represent standard errors (*n* = 3). **c** Subset of the MFuzz clusters from the clustering of all genes from the KMS time course with number of genes belonging to the indicated categories. Only genes strongly associated with a cluster’s predominant expression profile (membership value >0.5 as well as an FPKM >10 at the cluster’s expression peak) were selected which led to the capture of between a half and a third of each category

The number of induced genes that encode plant-polysaccharide degrading CAZymes (Fig. 4a), as well as their transcript abundance (Fig. 4b), increased sharply between the 3- and 6-h time points. The highest transcriptional abundances of plant-polysaccharide degrading CAZymes were generally found during the middle (9–24 h) time points, with the exception of a bi-modal gene transcript abundance pattern in the ILS time course that will be discussed later. This peak in transcript abundance of CAZyme-encoding genes was also demonstrated in clusters of gene transcript patterns generated using MFuzz [33] clustering for time-series data (Fig. 4c). Transcript abundance patterns were analysed in clusters of the KMS time course (Additional file 3: Table S2). The two most prominent clusters with regard to genes encoding biomass-degrading CAZymes were clusters 9 and 13 (Fig. 4c). Cluster 13 had a peak in expression at 9 h, while cluster 9, which contained by far the largest number of CAZy genes, showed a peak in expression at 24 h. Cluster 9 had more exo- and endocellulase-encoding genes than cluster 13 (5 compared to 1), and both clusters included genes predicted to encode proteins associated with hemicellulose- and pectin-degrading activities.

### Pretreatment influences transcript abundance of a subset of CAZy genes

To identify pretreatment-specific CAZy gene induction, we compared the plant-polysaccharide-active CAZymes that were relatively highly induced at least once during the time courses, as compared to repressive glucose conditions (i.e. with a DESeq *p*_*adj*_ < 0.05, FPKM of ≥50 on lignocellulose and log2 FC of ≥3).

**Fig. 5:**
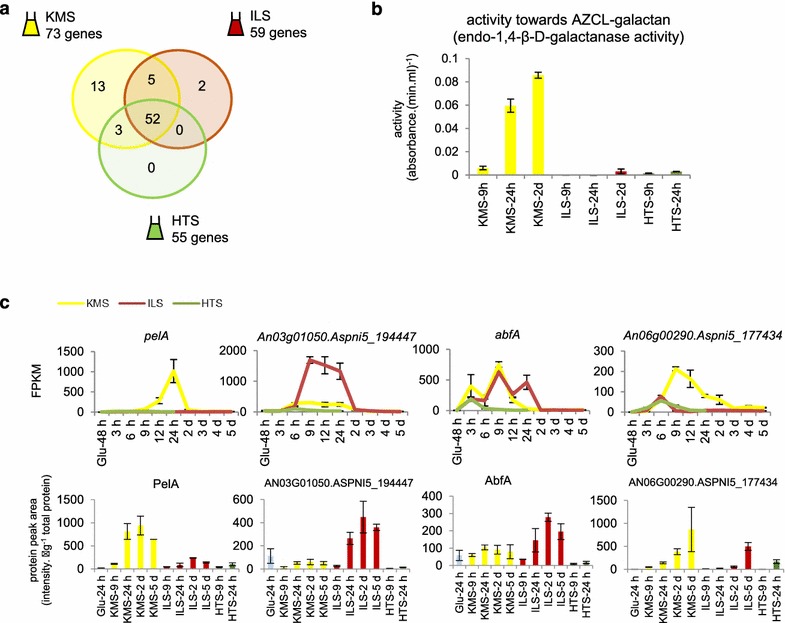
Pretreatment-specific CAZy gene expression. **a** Venn diagram comparing the lists of genes encoding plant-polysaccharide-active CAZymes that are induced (DESeq *p*_*adj*_ < 0.05, FPKM of ≥50 on lignocellulose and log2 FC of ≥3) on the different substrates (see Additional file 4: Table S3 for details on genes in different sections of the Venn diagrams), **b** β-(1,4)-endogalactanase activity detected on AZCL-galactan (potato). *Error bars* represent standard errors (*n* = 3), **c** The FPKM expression value and targeted proteomics value for a subset of the genes in the Venn diagram and their encoded proteins

The Venn diagram in Fig. 5a illustrates that a core set of 52 CAZy genes was induced on all substrates, while 23 genes seemed to have pretreatment-specific induction. The extent of pretreatment-specific gene expression seemed to be linked to the expected substrate complexity; KMS showed the highest number of specifically induced genes, while no genes were induced specifically on HTS.

The core set is composed of genes encoding many different enzymatic activities, such as cellulases, LPMOs, hemicellulases, some pectinases and pectin esterases (Additional file 4: Table S3). On the contrary, amongst the pretreatment-specific CAZy genes, the scope of activities was much more limited. While the endo-β(1,4)-mannanase-encoding gene *man5A* was induced specifically on KMS and HTS, the ILS-specific genes consisted of *aglB* and *mndA*, which encode exo-acting enzymes required for galactomannan degradation [34]. The set of genes whose induction was exclusive to KMS was highly enriched for genes encoding enzymes that are active on pectin (9/13 genes), especially the homo- and rhamnogalacturonan backbone (7/13). In particular, transcription of the endo-acting pectinases was highly abundant in KMS and almost completely lacking in ILS and HTS. Furthermore, the genes that were induced both on KMS and ILS, but not HTS, included 3 pectinolytic activity-encoding genes.

We further analysed genes encoding pectinolytic activity and found amongst the 58 genes encoding enzymes that (putatively) act on pectin [35]; other genes had similar expression trends with transcript levels most abundant on KMS or KMS and ILS (Additional file 4: Table S3). This included the β-1,4-endogalactanase-encoding gene (*galA An18g05940.Aspni5_187227*); assay data for β-1,4-endogalactanase activity correlated with the transcript abundance of the *galA* gene as the highest activity was measured from filtrates from the KMS cultures (Fig. 5b).

Four CAZy genes with particularly large differences in expression, and whose secreted protein levels correlated with their transcript level, further exemplify this trend (Fig. 5c). The *pelA* gene encodes a pectin lyase, induced by galacturonic acid [36, 37] and exclusively induced on KMS. An03g01050.Aspni5_194447 encodes a putative endo-β-1,6-galactanase [38] (sometimes annotated as a putative endoglucanase [13]), expression of which was induced exclusively on ILS and KMS. The gene *abfA* encodes an α-arabinofuranosidase active on side chains of pectin, which is induced by arabinose, xylose and polygalacturonic acid [35, 39, 40]. An06g00290.Aspni5_177434 encodes a putative β-galactosidase [41]. These last two genes were induced in early (3–6 h) time points on all substrates, but high transcription levels and protein amounts in middle (9–24 h) time points were only observed on KMS and ILS or KMS, respectively.

Taken together, we observed a trend where genes encoding for enzymes that degrade polymers (especially pectin) that were likely removed by the pretreatment showed a lack of induction or lower transcript abundance in the cultures with the corresponding pretreated substrates. While these trends associated with pretreatment, i.e. the presence of structural components, were generally consistent across multiple time points, the IL pretreatment also influenced transcript levels of a subset of genes in a manner that changed in time and resulted in a bi-modal transcript abundance pattern.

### Bi-modal CAZy gene transcript patterns exclusive to IL pretreatment

The increased digestibility of IL-pretreated substrates in the cultures and the corresponding accumulation of xylose and arabinose (Fig. 2) had a pronounced effect on the expression of CAZymes. The abundance of total CAZy transcripts in *A. niger* had a bi-modal pattern in the ILS cultures, whereas there was a mono-modal pattern in the untreated and HTS and KMS cultures (Fig. 4).

**Fig. 6:**
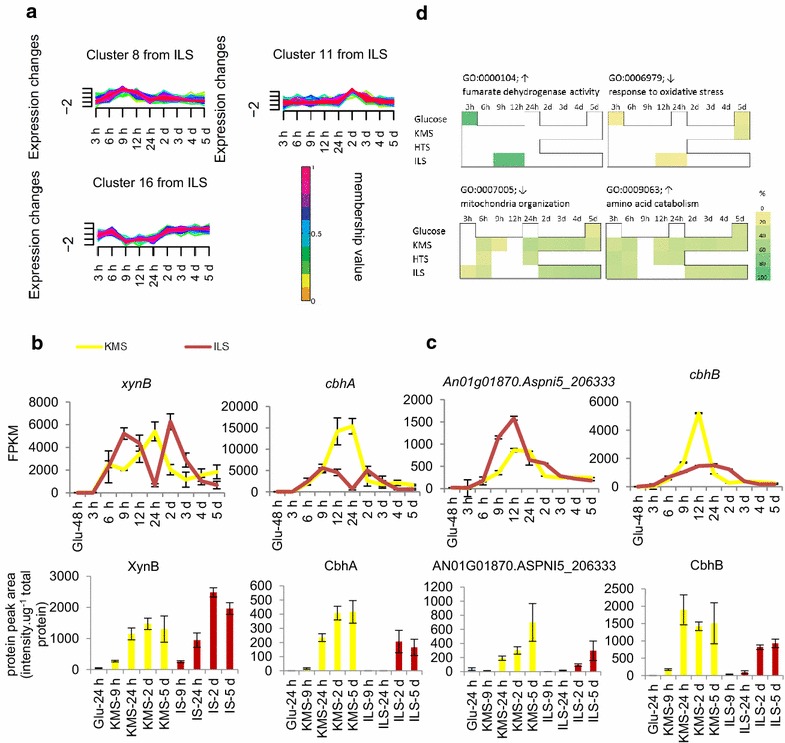
Suppression of gene expression on IL-pretreated substrates. **a** MFuzz clusters of SignalP annotated genes illustrate some of the clusters that included the expression pattern of interest (the membership value colour indicates how well an individual expression pattern in an MFuzz cluster fits the dominant pattern in that cluster), **b** the expression patterns of genes with bi-modal expression pattern showing the mean FPKM value and targeted proteomics values for the proteins encoded by these genes (*error bars* represent standard errors), **c** the expression patterns of genes without bi-modal expression pattern showing the mean FPKM value and targeted proteomics values for the proteins encoded by these genes (*error bars* represent standard errors) and **d** selection of GO terms either enriched or absent in both IL mid-time points and 3-h glucose

Correlation between high xylose and arabinose concentrations in the middle (9–24 h) IL time points and gene expression patterns was investigated using the Mfuzz clustering analysis of genes annotated as having a signal peptide. These genes encode the potential secretome, as well as some proteins that are deposited in membranes such as transporters [42]. Clusters were analysed from the ILS cultures’ time course that appeared to capture modulation in expression around time points where the sugar concentration was higher (clusters 1, 4, 6, 8, 10, 11, 13, 16, 17 and 20) (Fig. 6a; Additional file 5: Table S4).

We identified 63 genes whose pattern was bi-modal on ILS and mono-modal on KMS from the selected clusters from the ILS time course (Additional file 6: Table S5). Of these, 33 had a CAZy annotation and characterised CAZy genes with this pattern included *cbhA, eglA, axhA, xynB* and *xynA*, which were all found in cluster 8 from the ILS time course (Fig. 6b). Genes with this pattern included four uncharacterised genes annotated with the Pfam domain for sugar transporter (PF00083). Of the CAZy genes with the bi-modal pattern, 19 of these are regulated by the CAZyme-activating transcription factors XlnR or AraR based on the study of de Souza et al. [30] (Additional file 6: Table S5). Other genes did not display this bi-modality in transcript level pattern on ILS around the time points where the pentose sugar concentration was higher, such as *An01g01870.Aspni5_20633* and *cbhB* from clusters 5 and 9 from the ILS time course, respectively (Fig. 6c; Additional file 5: Table S4).

A subset of the proteins encoded by CAZy genes was analysed with targeted proteomics. Generally, there was no bi-modal pattern on the ILS compared to the KMS for the proteins whose genes had a bi-modal transcript level pattern. CbhA was not detected until a later time point on ILS compared to KMS, but otherwise the selected proteins whose genes had either mono-modal or bi-modal transcript level patterns had similar protein abundance profiles on KMS and ILS (Fig. 6b, c; Additional file 7: Figure S2). The lack of a correlation between the transcript levels and the targeted proteomics could be due to the accumulation of the secreted proteins without turnover (degradation) effects in the ILS cultures.

To help identify the cause of the observed bi-modal pattern, the gene expression in the KMS and ILS mid-time points (9–24 h) was analysed in more detail. The hierarchical clustering of CAZy gene transcription showed similarities in the transcript abundance patterns of ILS middle (9–24 h) time points to glucose-rich conditions (Fig. 3). The differentially expressed genes from these conditions compared to the Glu 48 h condition also shared enriched GO terms (Fig. 6d). Responses to oxidative and osmotic stress and glutamate metabolism were enriched in genes with reduced transcript levels. In the genes with increased transcripts, fumarate dehydrogenase activity was enriched, as were mitochondrial ATP synthesis coupled electron transport and coenzyme Q-cytochrome C reductase activity. The amino acid catabolism GO term was enriched in most conditions except for mainly the glucose-rich and IL middle (9–24 h) time points. For genes with reduced transcript levels, GO terms related to mitochondrial function were never enriched in the IL 9–24 h or the 3- and 24-h glucose time points, but were so in most lignocellulose conditions. This suggests that for the glucose 3 and 24 h and 9–24 h IL time points, high mitochondrial activity was required, for example, due to a higher growth rate, while for most other lignocellulose time points, mitochondrial activity was reduced.

### Genes encoding activities related to lignin breakdown show limited differential responses to the pretreated substrates

A subset of the auxiliary activity (AA) enzyme families is involved in lignin breakdown whereby those oxidoreductases utilise redox mechanisms [43]. *A. niger* has enzymes from six of these AA families (AA1, AA3, AA4, AA6, AA7 and AA8) but lacks the AA2 peroxidases which are essential for lignin depolymerisation but are only found in basidiomycetes [44]. The transcript abundance of individual AA encoding genes changed significantly between the Glu 48 h and lignocellulose cultures, while the total AA transcripts did not increase substantially (Additional file 8: Table S6). Overall, 13/57 AAs were induced on lignocellulose where 10/13 formed a core induced on all three substrates which included AA1, AA3, AA6 and AA7 members. The two AA1s in this core have been characterised as laccase-like multicopper oxidases (LMCOs) oxidising a range of aromatic compounds [45]. Other characterised AA1 members were either induced only on ILS (*mcoF*) or on both KMS and HTS (*mcoG*). Remarkably, between 3 and 6 h on the untreated and pretreated straws, one core-induced AA6 member (An01g05340.Aspni5_206038) was responsible for approximately half of the AA expression. For AA6s, 1,4-benzoquinone reductase is the only demonstrated activity [43] and the characterised fungal AA6s are all from basidiomycetes according to the CAZy website [46, 47]. This *A. niger* AA6 could have activity towards derivatives released from the lignin (which is composed of sub-units containing aromatic rings). Also, the transcript abundance of this AA6 correlated positively with the lignin content of the substrates with higher transcript abundance on KMS and HTS which had higher lignin contents compared to ILS, where both the transcript abundance and lignin contents were lower (Fig. 1a). Also noteworthy was a core-induced AA3 alcohol oxidase (An18g05480.Aspni5_42956) that differed remarkably in timing of induction between the pretreated (both 3 h) and the untreated (24 h) substrates. Analysis of the aromatics present in the culture filtrates could provide insight into the inducers of the AAs described here.

### Unannotated genes co-expressed with genes for plant-polysaccharide-degrading CAZymes include those encoding potentially novel enzymes

To identify genes encoding proteins with unknown functions that potentially could have a function during lignocellulose degradation, we investigated genes without Pfam domain annotation or with a Pfam domain of unknown function (DUF) whose transcription profile clustered with genes encoding predicted plant-polysaccharide-degrading CAZymes. In the KMS time course data, 4 MFuzz clusters contained 83% of selected CAZyme-encoding genes and 27 genes encoding uncharacterised proteins with a signal peptide. Similarly, 6 ILS and HTS clusters contained 72 and 79% of CAZyme-encoding genes, and 31 and 24 genes encoding uncharacterised proteins, respectively (Additional file 9: Table S7). In total, 5 genes were identified on all lignocellulosic substrates. The encoded proteins formed 4 main groups: 26 proteins with transmembrane domains, at least 9 proteins related to secretion stress or potential fungal cell wall proteins, a set of proteins for which no function or similarity could be derived, and 5 proteins, discussed in detail, similar to CAZymes.

Identified in KMS cluster 16, which contains >50% of genes encoding CAZymes, An16g00670.Aspni5_127791 showed increased expression on KMS from 9 to 24 h. A Phyre2 HMM-based search for structurally similar proteins [48] identified similarity to *B. subtilis* YuiC (PDB 4wjt), which cleaves chitin oligosaccharides, with conservation of the catalytic residue [49]. The mycoCLAP database for characterised lignocellulolytic enzymes [50] indicated similarity to the linker and chitosan-binding domain [51] of GH75 chitosanases. These results suggest that An16g00670.Aspni5_127791 may have chitin or chitosan binding/hydrolysis function.

The expression profile of An02g13300.Aspni5_173481 (Clusters KMS16, HTS13 and ILS8) is reminiscent of plant-polysaccharide-degrading CAZymes, with expression levels highest at 9–24 h on KMS (max ~120 FPKM) and ILS (max ~440 FPKM), matching the 9-h cluster induction peak. The encoded protein has similarity to putative GH43 members, although phylogenetic analysis and alignment with members from the most similar GH43 subfamilies [52] 17–22 indicated a lack of conserved residues. Phyre2 modelling indicated similarity to a galactose mutarotase, endoglucanase and α-mannosidase (PDB 2Q1N, 1CLC, 2WW1), albeit without conservation of catalytic residues.

Gene An13g02450.Aspni5_54830 (clusters KMS13 and ILS13) was moderately expressed on KMS 9-24 h, and induced to ~150 FPKM on ILS 9–12 h. The encoded protein has an IPR008928 domain, a six-hairpin glycosidase-like structure shared by a broad range of enzymes. An13g02450.Aspni5_54830 shares overall structural similarity with many of these enzymes with conservation of the catalytic acid residue, but it lacks key catalytic or characteristic residues of the most structurally similar rhamnosidases and disaccharide phosphorylases. The automated CAZyme annotator dbCAN [53] indicates that the protein belongs to GH121, a bacterial sequence family of which the only characterised enzyme liberates Ara*f*-β1, 2-Ara*f* from the glycan moiety of plant protein extension [54] and also has a IPR008928 domain. Together with the absence of gene expression on HTS, which lacks arabinose, this suggests a possible role of An13g02450.Aspni5_54830 in degrading arabinose-containing carbohydrates.

KMS12 and HTS10 clusters included An02g13630.Aspni5_37552, which is expressed under most conditions of lignocellulose degradation but only to ~30 FPKM. Its DUF159 domain is also found in a bacterial protein with multiple carbohydrate-binding modules, but while its structure suggests that it is a catalytic domain active on furanose sugars, no activity has been demonstrated so far [55]. Gene An02g11390.Aspni5_197780 (ILS14) was identified only in cluster ILS14, and high expression (to ~1100 FPKM) for all substrates was observed at the 3- and 6-h time point, but also on glucose at 3 h, suggesting a non-lignocellulose specific role. The Phyre2 structural model identified similarity to bacterial polysaccharide deacetylases with conservation of catalytic and other active site residues.

### Novel potential candidates for transcription factor-dependent engineering strategies for boosting CAZyme expression

The Zn_2_Cys_6_ transcription factors that play a major role in regulating biomass degradation were identified in the MFuzz KMS transcription profile clusters that had a peak in transcript abundance earlier than the clusters containing the biomass-degrading CAZy genes: *xlnR* (cluster 11) and *araR* (cluster 1) (Additional file 3: Table S2). Uncharacterised Zn_2_Cys_6_ transcription factors were also identified in clusters with characterised transcription factors or with CAZy genes, prompting more detailed analysis of transcription factor expression on all three of the lignocellulosic substrates using the MFuzz clustering of all genes datasets. Uncharacterised transcription factors that were annotated with at least one of four Pfam transcription factor domains were analysed to identify those that were putative activators of plant biomass-degrading CAZymes (Additional file 10: Table S8). The expression pattern of these transcription factors was analysed for patterns that followed the major trends in CAZyme expression as identified previously, via MFuzz clustering of transcription profiles. In total, 24 uncharacterised transcription factors were identified as putatively activating expression of the plant biomass-degrading CAZymes, and they also suggested ways to engineer *A. niger* to boost CAZyme production. Other transcription factors were discounted because annotations at AspGD suggested regulation of other aspects of fungal metabolism such as amino acids. 22 of the transcription factors are potential activators of the CAZymes induced between 6 and 24 h on the untreated and pretreated straws.

16 of the transcription factors may engage in the regulated expression of the CAZymes that are repressed in the presence of the higher pentose sugar concentrations on ILS as they had remarkably similar expression profiles to the repressed CAZymes. A role in activation of the pectinases that are specifically induced on KMS is suggested for An14g05670.Aspni5_41900 by the higher and longer induction on KMS, possibly in addition to or modifying the action of recently identified regulators of genes encoding pectinolytic enzymes, such as RhaR [56] and GaaR [57]. The recently discovered transcriptional activator and repressor duo GaaR–GaaX [58] shares the expression profile of higher induction on KMS.

An04g09790.Aspni5_214859 could be the activator of some of the later induced CAZymes, and it was found in the same MFuzz transcription cluster (KMSall_genes_cluster 5) as a later induced GH28 pectinase An01g14650.Aspni5_172236. In Additional file 10: Table S8, other uncharacterised plant biomass-degrading CAZymes that clustered with the 24 transcription factors are listed. Three of the transcription factors (An16g01390.Aspni5_130681, An16g02920.Aspni5_53646 and An11g04160.Aspni5_178503) are annotated as being similar to AmyR which in *A. niger* was shown to have a broader regulatory role beyond maltose regulation [59], and this dataset supports a broad role for these three transcription factors.

The putative activating roles of the 24 transcription factors suggested engineering strategies to increase the production of CAZymes. Overexpression of the transcription factors that are repressed on ILS 9–24 h could drive the transcription of the CAZymes repressed in the presence of higher pentose sugar concentrations. Overexpression of An14g05670.Aspni5_41900, which is a putative activator of the pectinases induced on KMS, could be used to drive the transcription of these pectinases on the pretreated substrates.

### Temporally limited transcript abundance patterns highlight the utility of a broad time course

**Fig. 7:**
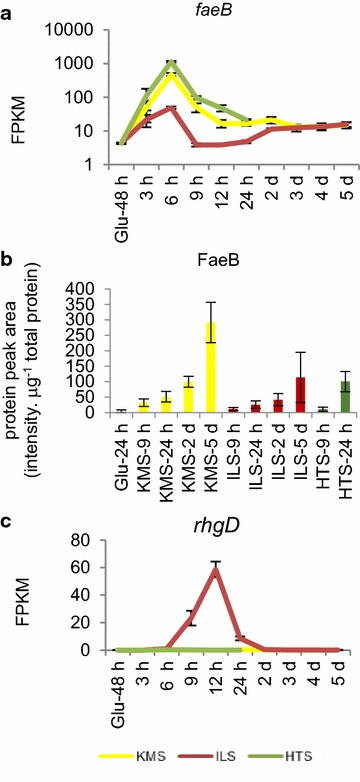
Temporally limited transcript abundance patterns. **a** *faeB* transcript abundance pattern, **b** targeted proteomics values for the FaeB protein showing that, although the period of *faeB* induction is short, FaeB persists for much longer in the cultures and **c** *rhgD* transcript abundance pattern. *Error bars* represent standard errors

A broad time course afforded the opportunity to identify genes that had increased transcript abundance at a small subset of the time points. Two genes of particular interest were identified that had pretreatment-dependent increases in transcript abundance compared to the Glu 48 h control in a subset of the time points (Fig. 7). The gene *faeB*, which encodes a feruloyl esterase and is induced by aromatic compounds, but not sugars [31], had transcript abundance at least tenfold higher on the untreated and HT-pretreated straw compared to the IL-pretreated straw at 6 h (Fig. 7a). Although transcript levels from *faeB* were increased for a relatively short time, FaeB persisted in the cultures for at least 5 days (Fig. 7b). The differences in protein abundance between the IL cultures and untreated or HT-pretreated were smaller than the differences in transcripts suggesting there may be a peak in *faeB* expression at time points other than those measured. One possible reason for the lower measured *faeB* transcript level on the IL-pretreated substrates could be that the inducing aromatics have been partially or fully removed by the IL pretreatment. de Vries et al. [31] showed that different aromatic compounds can induce *faeB* to varying levels and there are strong similarities of these aromatic compounds with the compounds that Varanasi et al. [60] showed are released from lignocellulose using an IL pretreatment similar to that used in this study. The other gene *rhgD* (*An11g06320.Aspni5_178393*) had increased transcript abundance around the 12-h time point on the IL-pretreated straw cultures (Fig. 7c). Martens-Uzunova and Schaap [35] annotated *rhgD* as a putative rhamnogalacturonase but did not detect any expression of *rhgD* in their experimental conditions that included growth on pectin and pectin components. The particular concentrations of sugars in the mid-IL time point cultures could cause the increased transcript abundance of *rhgD*.

## Discussion

Differences in compositional and structural features of the substrate for fungal cultivation, caused by pretreatment, can be linked directly to changes in transcript abundance, protein levels and enzyme activity.

HT pretreatment of the wheat straw has likely resulted in loss of pectin, which in the KMS cultures forms the basis of induction, and high levels of transcripts, for the genes encoding the pectinolytic enzymes. Removal of pectin and some hemicelluloses by HT pretreatments but not by IL pretreatments is supported by the presence of at least tenfold greater concentrations of galactose and arabinose in the acid hydrolysates of the KMS and ILS substrates compared to the HTS substrate (Fig. 1a). Galactose and arabinose are prominent components of hemicelluloses and pectin, and either proven or potential inducers of a set of genes with higher transcript levels on KMS and ILS compared to HTS. The galacturonic acid (from the backbone of pectin) content was not measured in the untreated or pretreated substrates but the transcript abundance pattern of a subset of pectinolytic genes including *pelA,* which galacturonic acid induces, suggests that the HT and IL pretreatment did not retain the homogalacturonan pectin. This is in agreement with the reported complete loss of homogalacturonan epitopes but not arabinogalactan epitopes in IL-pretreated switchgrass [61]. However, it should be noted that not all genes encoding pectinolytic enzymes followed the same expression profile, suggesting different sensitivity to inducers, and/or reflecting the fact that subsets of pectinolytic genes are modulated by the presence of arabinose, rhamnose and ferulic acid [39, 57].

The increased digestibility of IL-pretreated wheat straw (Fig. 2b), which is the result of a decrease in complexity of the interconnections between carbohydrate polymers [7], resulted in an accumulation of pentose sugars (Fig. 2c) suggesting a carbon excess of what was required for the rate of growth. This was coupled with dramatic changes in transcript levels in the ILS cultures at the mid-time points (9–24 h) to patterns more similar to the glucose cultures (Figs. 3, 6d). However, a subset of genes showed increased transcript levels or exclusive induction of expression on ILS, indicating that these genes could be positively regulated by the higher concentrations or ratio of concentrations of small carbohydrates. Furthermore, these genes without the bi-modal pattern are candidates to investigate whether their activities could be better adapted to saccharifying the polymers and linkages that the IL-pretreated straw presents to the fungus at these middle (9–24 h) time points.

A range of genes encoding uncharacterised proteins were found to cluster with genes encoding plant-polysaccharide-degrading CAZymes. The identified proteins with transmembrane domains could be involved in sensing or signalling during lignocellulose degradation, but for most a lack of clear homology to functionally characterised proteins inhibits functional prediction; gene deletion or overexpression studies would be required to determine their roles. The identification of 5 genes that could possibly encode novel CAZymes proves the value of detailed (structural) homology analysis. Based on the identified homology to characterised proteins, An16g00670.Aspni5_127791 may encode a chitinase or chitosanase that may be involved in fungal cell wall remodelling during exposure to lignocellulose. While no functional prediction could be made for An02g13630.Aspni5_37552 and An02g13300.Aspni5_173481, the identified homology and gene transcription profile of An13g02450.Aspni5_54830 suggest a potential role in the degradation of arabinose-containing carbohydrates. Follow-up research based on homo- or heterologous production of these enzymes and subsequent biochemical characterisation will reveal if these predictions correctly identified potential novel CAZymes, and if so whether they have improved catalytic efficiency or novel substrate–product spectra that warrant application in enzyme mixtures for lignocellulose saccharification.

With regard to engineering a fungus that might be better adapted to CAZyme production from lignocellulose, the induced AAs such as the AA6 are excellent candidates for overexpression in order to reduce growth-inhibiting effects of lignin-derived aromatics. Other engineering approaches can include modification of transcription factor expression; a set of 24 potential transcriptional activators of CAZyme-encoding genes have been identified in this study. Follow-up analysis, e.g. through gene deletion or overexpression strains, to identify the CAZymes (if any) that are regulated by these transcription factors, would be instrumental in supporting the design of engineering strategies. Other candidates for engineering strategies amongst the oxidoreductases may not have been identified because they are subject to complex regulation through multiple repressors (e.g. excess carbon and nitrogen) and multiple activators (e.g. metal co-factors and aromatics) [62]. The excess nitrogen present in the cultures may explain a lack of induction for some of the AAs in this study.

Pretreatments function to make a substrate more digestible, but surprisingly we were able to extract RNA from later time points from KMS cultures compared to HTS cultures, suggesting that the *A. niger* cultures were able to remain viable for longer periods on the untreated straw (albeit to a lesser extent than the IL-pretreated straw) than the HT-pretreated straw. Concurring with this, autolysis-related genes such as *chiB* and the sporulation regulator *brlA* (indicators of fungal starvation stress) were induced earlier in the HTS compared to the KMS or ILS cultures (Additional file 7: Table S5). The lesser viability of *A. niger* on the HT-pretreated straw compared to the IL-pretreated straw could be explained by the retention of many of the hemicelluloses by the IL pretreatment (which are lost as part of the HT pretreatment) and the decrease in cellulose crystallinity by the IL pretreatment. A deficiency of some essential nutrients in the HT-pretreated straw could also be a factor. Understanding the reasons for the poorer performance on the HT-pretreated straw could then allow for greater saccharification and better understanding of the fungal degradation from a longer time course. It is notable that of the complex substrates used in this study, the substrate on which *A. niger* was able to remain viable—as indicated by RNA extraction—for the shortest amount of time (i.e. HT pretreated), is the most different from what *A. niger* has evolved as a saprobe to grow on in nature.

IL-pretreated straw could function as an inducer for CAZyme production in a biorefinery but not necessarily the HT-pretreated straw as understanding of the reasons for the poorer performance on the HT-pretreated straw is required. Exposure of the fungus to a greater range of concentration of sugar inducers could lead to induction of transcription of CAZymes such as the *rhgD* pectinase that may not otherwise be induced. The suppression of CAZy transcripts that occurred on the IL-pretreated substrate could be attenuated by the use of CCR derepressed strains as the suppression here is likely mediated via CreA. Moreover, tolerance to ionic liquids is considered a general property of the Aspergilli [63] which could mean that residual ionic liquid leftover from the pretreatment may not be inhibitory to *A. niger*.

## Conclusions

This study demonstrates that time and pretreatment each have a major influence on the fungal responses to lignocellulose. We highlighted that the transcript levels in *A. niger* correlated with the changes in substrate composition and properties brought about by the pretreatments. This study emphasises the value of broad time courses when studying fungal responses to lignocellulose to identify the roles of induction and repression in the levels of transcripts particularly in response to pretreated substrates. Ultimately, improved understanding of the responses will aid identification of strategies to improve the efficiency of lignocellulose saccharification and subsequent production of a biofuel. Key features identified in this study contribute to understanding, such as the potential of IL-pretreated substrates to induce high CAZyme expression when CCR is abolished, or are suitable candidates for future research in this field, such as putative new regulators to boost CAZyme production and the potential novel CAZymes that could have novel substrate or product specificities.

## Methods

### Substrates and pretreatments

Wheat straw (cv. Cordiale) was ground with a Fritsch Pulverisette 19 Universal Cutting Mill (Fritsch, Germany) fitted first with a 2 mm sieve. This ground material was passed again through the cutting mill fitted on the second time with a 0.5-mm sieve. The ionic liquid (IL) pretreatment was performed using the chemical 1-ethyl-3-methylimidazolium acetate ([C2mim][OAc]) (Sigma-Aldrich) based on a published method [64]. The details of the ionic liquid pretreatment are as follows: A slurry of 10% biomass solids in the IL was mixed thoroughly and then incubated for 2 h at 160 °C in a glass beaker in an atmospheric oven. The resulting gel-like material was cast as a thin film on a flat surface, soaked in water to regenerate the solid and rinsed multiple times in water over seven days to remove all water-soluble products. The hydrated film was air-dried overnight and oven-dried for 2 h at 100 °C before measurement of the final dry mass. The hydrothermal treatment was performed according to a published method [6]. The details of the treatment are as follows: A slurry of biomass in water was made up at 20% solids (1:4 LR), which was sealed into a steel reactor tube and then placed in an oven preheated to 200 °C. Tubes were held in the oven for 2 h, with the internal sample temperature following a ballistic heating profile. The tubes were cooled by running tap water and the contents were then transferred and filtered through a Whatman No. 1 filter paper, washed multiple times with water, air-dried overnight and oven-dried at 100 °C for 2 h, prior to measurement of the final dry mass. Additional file 11: Figure S3 contains representative images of the particle sizes of the untreated and pretreated straw substrates.

### Compositional and structural analysis of substrates

A total acid hydrolysis assay of the wheat straw was carried out for determination of constituent monosaccharides. 30 mg of dry sample was immersed in 1 ml 12 M sulphuric acid for 2 h at 35 °C in a capped Pyrex tube, then diluted to 1 M acid by addition of 11 ml of deionised water and followed by incubation for 2 h at 98 °C [6]. Analysis of soluble sugar monomers in the hydrolysate was carried out by high-performance anion exchange chromatography with pulsed amperometric detection (HPAEC-PAD) (Dionex, UK), using a CarboPac PA20 column under isocratic conditions, with 10 mM NaOH as the mobile phase at a working flow rate of 0.5 ml min^−1^. Glucose, xylose, arabinose and galactose were used as standards with mannitol as internal standard. In terms of sensitivity of this analysis, after dilution to accommodate the dynamic range of the instrument, the detection limit for a monosaccharide was ~0.5% w/w of the total biomass solids. Lignin content was assessed via acetyl bromide determination. In short, 100 mg biomass was added to 4 ml 25% acetyl bromide in glacial acetic acid and incubated at 50 °C for 2 h with occasional shaking. 12 ml glacial acetic acid was added and particulates removed by centrifugation. To 0.5 ml supernatant, 2.5 ml glacial acetic acid and 1.5 ml of 0.3 M sodium hydroxide were added, followed by 0.5 ml of 0.5 M hydroxylamine hydrochloride and a further 5 ml glacial acetic acid, after which the optical density was determined at 280 nm. Acetyl bromide-treated lignin (Sigma) in 80% (w/v) aqueous dioxane was used as a standard. Wide-angle X-ray diffractograms of untreated biomass and pretreated substrates were measured using a Siemens S5000 system with a vertical theta–theta goniometer, as described previously [65]. An amorphous subtraction procedure was carried out using the diffractograms of dry ball-milled samples from each specific biomass species. The percentage crystallinity was determined from the crystalline integral after subtraction of the underlying amorphous profile, as a proportion of the total integral. An estimation of the lateral dimensions of the cellulose fibrils in the cell walls was made using the Scherrer equation (*L* = 0.9*λ*/(IB cos(θ))), where L is the dimension corresponding to the cellulose-I [200] reflection; IB is the [200] integral breadth, following curve fitting; *λ* is the X-ray wavelength (Cu Kα = 1.542 nm) and θ is the diffraction angle of the [200] peak in radians.

### Measurement of residual xylan in shake-flask cultures

The solids from 24 h and 5 d shake-flask cultures (where available) were recovered by centrifugation in 50-ml tubes for 10 min at 3200 *g*, then freeze-dried before weighing. 30 mg of the freeze-dried solids was acid-hydrolysed (as described elsewhere), and the xylose in the hydrolysates was quantified with a xylose assay kit (K-XYLOSE) (Megazyme). The amount of xylan present was calculated by dividing the amount of xylose by 0.88, the anhydrous correction factor. The total amount of residual xylan in the shake-flask culture was calculated by then dividing by the fraction that the 30 mg analysed was of the total mass of freeze-dried solids recovered from the flask (i.e. the fraction = 30 mg/mass of solids recovered). Xylose was quantified to estimate the residual lignocellulose as xylose is not a component of the fungal cell wall.

### Saccharification assays

The saccharifying enzyme mixture of Celluclast (Sigma) and Novozyme 188 (Sigma) was prepared as a 1:1 mixture. 30 mg of sample was saccharified with an enzyme loading of 6 EGU (based on the manufacturer’s measurement) (~0.6 FPU) per 30 mg of sample in 50 mM citrate buffer pH 4.8 with 0.02% w/v NaN_3_ in a total volume of 1.5 ml for 72 h at 50 °C with shaking. The reactions for each of the untreated and pretreated substrates were performed in triplicate. After 72 h, the glucose released was quantified using the GOPOD assay kit (K-GLUC) (Megazyme) and the xylose with a xylose assay kit (K-XYLOSE) (Megazyme) and expressed per mg of initial sample. As a control, Whatman filter paper was saccharified and the saccharification efficiency was >90%. Corrections were made for any sugars present in the enzymes mixture or substrates controls where no enzymes were added.

### Culture conditions

The culture conditions were described previously with the pH of the Aspergillus minimal media (AMM) set to 6.5 with NaOH [9]. Briefly, 1% w/v glucose cultures were inoculated with *A. niger* N402 spores at a final concentration of 1 × 10^6^ ml^−1^ and incubated at 28 °C for 48 h. The mycelia were collected by filtering through Miracloth, and 1.5 g wet weight was transferred to flasks containing one of the following carbon sources at a 1% w/v final concentration in AMM: glucose, untreated straw, ionic liquid-pretreated straw or hydrothermally pretreated straw. The cultures were incubated for the times shown in Fig. 2a. The lignocellulose substrates were autoclaved in AMM at 1% w/v concentration for sterilisation purposes. The cultures were incubated on three consecutive weeks with one of the three replicates for each condition in each of the weeks. Mycelia were collected for RNA extraction, and the filtrate of the culture was flash-frozen in liquid nitrogen and stored at −80 °C for subsequent sugar and proteomics analyses.

### RNA extraction

RNA was extracted with Trizol (Invitrogen), as described previously [9] and the RNA was further purified and DNAse treated on-column with the NucleoSpin RNA II Kit (Machery-Nagel, Germany) according to the manufacturer’s instructions.

### RNA sequencing

Plate-based RNA sample prep was performed on the PerkinElmer Sciclone NGS robotic liquid handling system using Illumina’s TruSeq Stranded mRNA HT sample prep kit utilising poly-A selection of mRNA following the protocol outlined by Illumina in their user guide: http://support.illumina.com/sequencing/sequencing_kits/truseq_stranded_mrna_ht_sample_prep_kit.html, and with the following conditions: total RNA starting material was 1 μg per sample and 10 cycles of PCR was used for library amplification. The prepared libraries were then quantified using KAPA Biosystem’s next-generation sequencing library qPCR kit and run on a Roche LightCycler 480 real-time PCR instrument. The quantified libraries were then multiplexed into pools of 5 or 6 libraries each, and the pool was then prepared for sequencing on the Illumina HiSeq sequencing platform utilising a TruSeq paired-end cluster kit, v3, and Illumina’s cBot instrument to generate a clustered flowcell for sequencing. Sequencing of the flowcell was performed on the Illumina HiSeq2000 sequencer using a TruSeq SBS sequencing kit, v3, following a 2 × 100 indexed run recipe.

### Mapping of RNA-seq reads and statistical analysis

Reads were mapped to a reference genome, which used as its base the genome sequence and annotation of the *A. niger* ATCC 1015 strain (Aspni5 - http://genome.jgi.doe.gov/Aspni5/Aspni5.home.html) [28]. The reference genome was subsequently annotated with genes annotated in the *A. niger* 513.88 CBS strain [27] that Andersen et al. [28] identified as ‘missing in ATCC’ in their comparative analysis of the annotations of the two strains. Raw reads from each library were aligned to the reference genome using TopHat [66] with only unique mapping allowed. If a read mapped to more than one location, it was ignored. FeatureCounts [67] was used to generate the raw gene counts. DESeq2 (version 1.2.10) [68] was subsequently used to determine which genes were differentially expressed between pairs of conditions. The parameters used to call a gene DE between conditions were *p*_adj_ value < 0.05. Additional file 12: Table S9 contains the FPKM values for each sample cultures with an untreated or pretreated straw substrate, as well as the DESeq2 analysis for the comparison of each condition with the Glu 48 h control.

### Clustering analyses of gene expression patterns

Hierarchical clustering of the 27 conditions was conducted with the function heatmap2 within the R package gplots using the gene expression values (FPKM), which were first log-transformed and then quartile-normalised. Only genes with an FPKM value >1 in at least one of the conditions were included. C-means clustering of the normalised gene expression values (FPKM) over time was calculated using the R package Mfuzz [33]. The c-means clustering was performed on the subset of genes annotated with a signal peptide as well as all the genes. The optimal range of clusters was first explored for each of the time courses using the corresponding Mfuzz function “cselection”. Clustering of genes based on these range values was then conducted, and the genes within each cluster were determined. The gene content and associated data of the Mfuzz clusters used for subsequent analysis are given in Additional file 4: Table S3, and for genes annotated with a signal peptide and for all genes from the KMS time course in Additional file 3: Table S2, for all genes from the ILS time-course in Additional file 13: Table S10 in and for all genes from the HTS time course in Additional file 14: Table S11. For the clustering analysis, the annotation of genes with a signal peptide by Braaksma et al. [42] was used, and for CAZy annotations the annotations for *A. niger* from www.cazy.org (from February 2015) were used.

### Gene ontology (GO) enrichment analysis

Gene Ontology (GO) enrichment analysis of each set of selected genes was performed using the Bioconductor package GOseq (v1.18.0) [69]. GO annotations for the genes were from the FetGOat resource (http://www.broadinstitute.org/fetgoat/) [70]. The GO annotations for the ATCC 1015 gene model and the CBS 513.88 gene model were used and combined to make a non-redundant list of GO annotations for a particular gene. The input for the enrichment analysis was the lists of significantly different genes from the DESeq2 analysis that had a log2 fold change as calculated by DESeq2 of ≤ −1 or ≥1 and a *p*_adj_ ≤ 0.05. Genes with an FPKM < 1 in both conditions being compared were excluded. The DESeq2 lists from the comparison of the Glu 48 h control with each of the other 26 conditions were used.

### Monosaccharide analysis of culture filtrates

The culture filtrates were heated at 100 °C for 5 min to inactivate enzymes in the culture filtrate that could degrade the sugars. The culture filtrates were analysed using HPAEC-PAD with a Dionex ICS-3000 Ion Chromatography System System (Dionex, UK) using a CarboPac PA20 column with a 10 mM NaOH isocratic system at a working flow rate of 0.5 ml min^−1^ at 30 °C. Glucose, xylose, arabinose and galactose were used as standards.

### Targeted proteomics

For the targeted proteomics analysis, culture filtrates for selected samples were thawed and cOmplete (EDTA-free) protease inhibitors (Roche) were added. The culture filtrates were concentrated using VIVAspin columns (5000 MWCO) (GE life sciences) at 4°C to ~10–20 times the initial concentration. The proteins in the concentrated sample were precipitated using trichloroacetic acid, and the protein pellet was washed several times with acetone before air-drying. Samples were analysed using an Agilent 1290 liquid chromatography system coupled to an Agilent 6460 mass spectrometer (Agilent Technologies; Santa Clara, CA). Peptide samples were loaded and separated on a Ascentis Express Peptide C18 column (5 cm length ×2.1 mm ID, 2.7 µm particle size) (Sigma-Aldrich; St. Louis, MO) operating at 60 °C operated at a 400 µl min^−1^ flow rate. A 10-min method with the following gradient was used: 98% Buffer A (0.1% formic acid) and 2% Buffer B (99.9% acetonitrile, 0.1% formic acid) were held for 2 min; then B was increased to 10% in 30 s, followed by an increase to 40% B over 3.5 min. Buffer B was increased to 90% in 30 s, where it was held for 2 min, then decreased to 2% B over 30 s, where it was held for 1 min to re-equilibrate the column to the starting conditions. Peptides were introduced into the mass spectrometer from the LC, using an Agilent Jet Stream Electrospray Ionization source (Agilent Technologies) operating in positive ion mode. Source parameters that were used include gas temperature (250 °C), gas flow (13 l min^−1^), nebulizer (35 psi), sheath gas temp (250 °C), sheath gas flow (11 l min^−1^) and VCap (3500 V). The data were acquired with Agilent MassHunter Workstation Software, LC/MS Data Acquisition B.06.00 (Build 6.0.6025.3 SP3). Skyline version 3.1.0.7382 software (MacCoss Lab, University of Washington) was used to determine 2–4 peptides per protein and optimal SRM transitions (3–5 transitions per peptide). Quantification of the SRM peak areas was processed using the mProphet algorithm [71] that is part of Skyline. Only peaks that passed a 0.05 *q* value cut-off were quantified and analysed further.

### Enzyme assays

For the enzyme assays with AZCL-Galactan (Potato) (I-AZGLP), appropriate volumes of the concentrated culture filtrates were incubated for 3 h at 28 °C with 150 rpm shaking with 1% w/v concentration of the dyed substrate in 100 mM sodium acetate pH 4.5 in a total volume of 250 μl in 96-well plates. Reactions were stopped with 2% Trizma base as per manufacturer’s instructions, and absorbance was measured at 590 nm with a plate reader.

## Additional files

**Additional file 1: Figure S1.** Heatmap of hierarchical clustering by condition to illustrate the relationships between conditions. The conditions were clustered using the log transformed and quartile normalised mean FPKM values of SignalP annotated genes. Genes whose expression was not ≥ 1 FPKM in any of the time points on any media were not clustered.

**Additional file 2: Table S1.** pH of culture filtrates. The pH of the culture filtrates is shown as the mean of the biological replicate flasks with standard errors.

**Additional file 3: Table S2.** CAZy, transcription factor and sugar transporter content of MFuzz clusters from the KMS time-course. File with lists of genes from each cluster from the KMS time-course of all genes from the categories of CAZy, Pfam for sugar transporters and Pfam for Zn_2_Cys_6_ transcription factors that met selection criteria. Only genes strongly associated with a cluster’s predominant expression profile (membership value > 0.5 as well as an FPKM > 10 at the cluster’s expression peak) were selected which led to the capture of between a half and a third of each category.

**Additional file 4: Table S3.** Genes in Venn diagram and pectin related gene expression. The file contains lists, identity and expression values of the genes in different parts of the Venn diagram in Fig. 5a and the expression patterns of the genes annotated by Martens-Uzunova and Schaap [35] acting on pectin. Induced was considered as significantly higher transcript abundance (DESeq *p*_*adj*_ < 0.05 log2 FC of ≥ 3) on lignocellulose compared to the glucose control, along with FPKM of ≥ 50 on lignocellulose.

**Additional file 5: Table S4.** MFuzz clusters of SignalP annotated genes. File with lists of genes in different clusters in clustering analysis of SignalP annotated genes. The file lists the genes in each of the clusters from each of the time-courses. The file also contains images of the MFuzz cluster set for each of the time-courses.

**Additional file 6: Table S5.** SignalP annotated genes with bi-modal expression pattern. File with the genes (annotated as having a signal peptide) that were identified as generally having a bi-modal pattern on ILS and a mono-modal pattern on KMS. The dataset of de Souza et al. [30] was used to indicate whether the CAZy genes with the bi-modal expression pattern were regulated by XlnR and/or AraR transcription factors.

**Additional file 7: Figure S2.** Graphs of the targeted proteomics data related to the gene repression patterns on the IL pretreated substrate.

**Additional file 8: Table S6.** Analysis of genes encoding enzymes with auxiliary activities (AA) relevant to lignin breakdown. Graphs of the total FPKM attributed to AA families relevant to lignin breakdown, number of induced AA members in each of the conditions compared to the Glu-48 h control, Venn diagram comparing the induced genes on each of the time courses and the dataset of the AA FPKM values and Venn diagram membership of the induced AAs.

**Additional file 9: Table S7.** Analysis of genes encoding proteins with unannotated functions. Analysis of the genes without a Pfam annotation or with only Pfam domains of unknown function whose transcription profiles co-cluster with genes encoding plant-polysaccharide active CAZymes in the MFuzz clustering of SignalP annotated genes dataset (see Additional file 5, Table S4).

**Additional file 10: Table S8.** Candidates for transcription factor-dependent CAZyme expression engineering strategies. The table lists uncharacterised transcription factors whose expression positively correlates with CAZyme expression, categorises their expression patterns and suggests reason(s) for inclusion in an engineering strategy to boost CAZyme expression. An image of the graphs of the expression pattern for each transcription factor is included. Uncharacterised plant biomass degrading CAZymes that clustered with the transcription factors in the MFuzz clustering of all genes are listed.

**Additional file 11: Figure S3.** Particle sizes of untreated and pretreated lignocellulosic substrates. The figure contains images of the particle sizes of the untreated and pretreated straw substrates. The yellow scale bar in each image represents a length of 10 mm.

**Additional file 12: Table S9.** File with FPKM values and DESeq2 statistics for all samples. The file contains gene identifiers and various annotations along with the FPKM values for each sample and condition, the fold change and *p*_adj_ values as calculated by DESeq2 for the comparison between the Glu 48 h control and the other 26 conditions.

**Additional file 13: Table S10.** CAZy, transcription factor and sugar transporter content of MFuzz clusters from the ILS time-course. File with lists of genes from each cluster from the ILS time-course of all genes from the categories of CAZy, Pfam for sugar transporters and Pfam for transcription factors that met selection criteria. Only genes strongly associated with a cluster’s predominant expression profile (membership value > 0.5 as well as an FPKM > 10 at the cluster’s expression peak) were selected which led to the capture of between a half and a third of each category.

**Additional file 14: Table S11.** CAZy, transcription factor and sugar transporter content of MFuzz clusters from the HTS time-course. File with lists of genes from each cluster from the HTS time-course of all genes from the categories of CAZy, Pfam for sugar transporters and Pfam for transcription factors that met selection criteria. Only genes strongly associated with a cluster’s predominant expression profile (membership value > 0.5 as well as an FPKM > 10 at the cluster’s expression peak) were selected which led to the capture of between a half and a third of each category.

## Abbreviations

CAZy: carbohydrate-active enzymes
EGU: endoglucanase unit
FPKM: fragments per kilobase of gene model per million mapped fragments
GO: gene ontology
HPAEC-PAD: high-performance anion exchange chromatography with pulsed amperometric detection
HT: hydrothermal
HTS: hydrothermally pretreated straw
IL: ionic liquid
ILS: ionic liquid-pretreated straw
KMS: knife-milled (untreated) straw
MWCO: molecular weight cut-off
*p*_adj_: *p* value from DESeq statistical analysis adjusted for multiple hypothesis testing
SignalP: signal peptide
TCA: tricarboxylic acid
