# Supplementary figures and images for: Expression of Aspergillus niger CAZymes is determined by compositional changes in wheat straw generated by hydrothermal or ionic liquid pretreatments
Source: Biotechnol Biofuels. 2017 Feb 7;10:35. doi: 10.1186/s13068-017-0700-9 (PMC5294722; doi:10.1186/s13068-017-0700-9)

Clustering of conditions using FPKM  
values of SignalP annotated genes

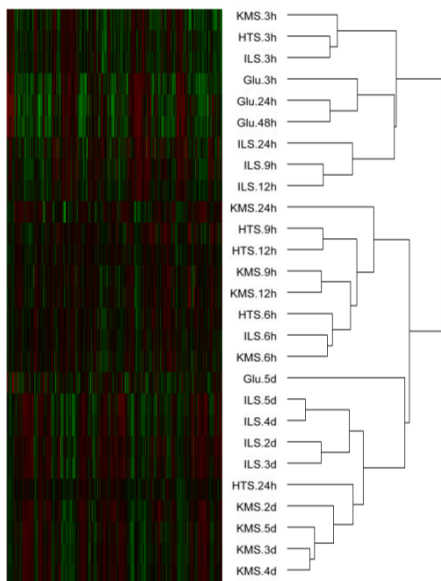

Supplement: Supplementary file 1 — Additional file 1: Figure S1. Heatmap of hierarchical clustering by condition to illustrate the relationships between conditions. The conditions were clustered using the log transformed and quartile normalised mean FPKM values of SignalP annotated genes. Genes whose expression was not ≥ 1 FPKM in any of the time points on any media were not clustered. [file 13068_2017_700_MOESM1_ESM.pdf]

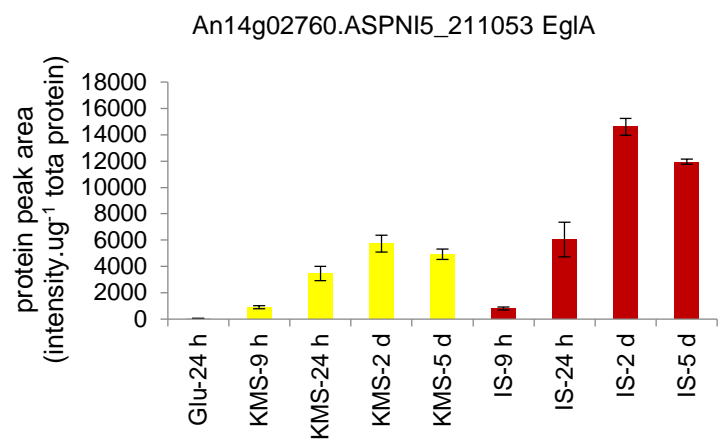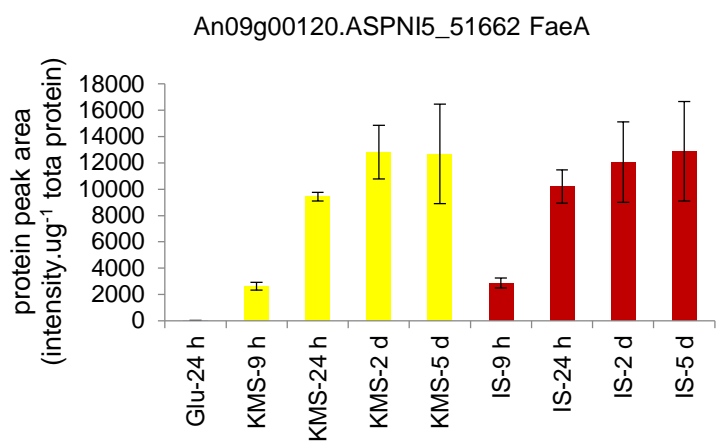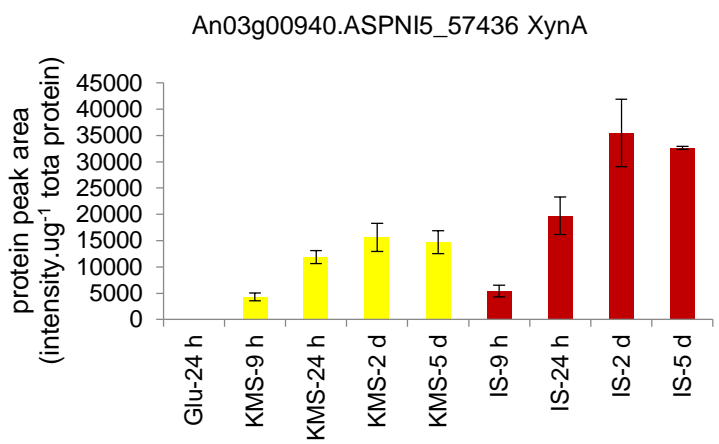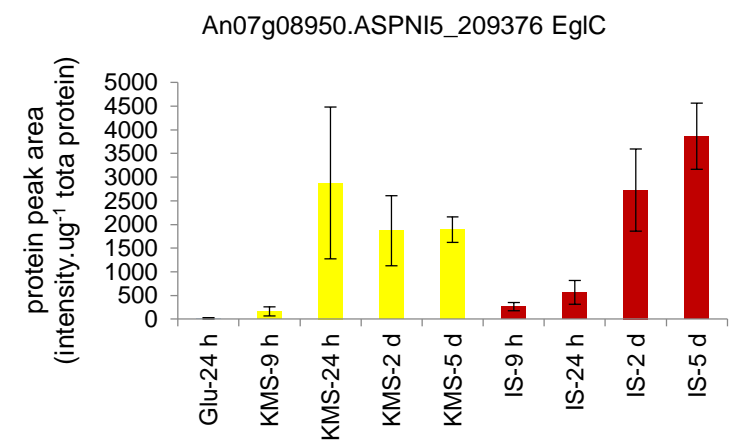

Supplement: Supplementary file 7 — Additional file 7: Figure S2. Graphs of the targeted proteomics data related to the gene repression patterns on the IL pretreated substrate. [file 13068_2017_700_MOESM7_ESM.pdf]

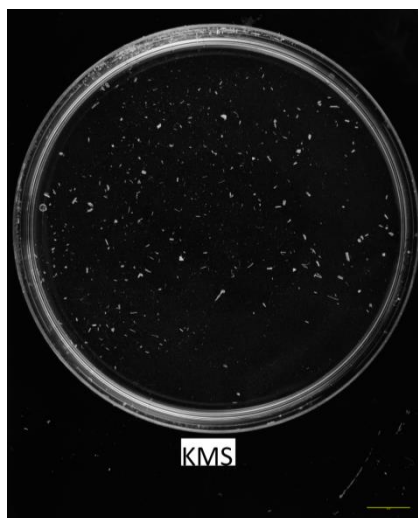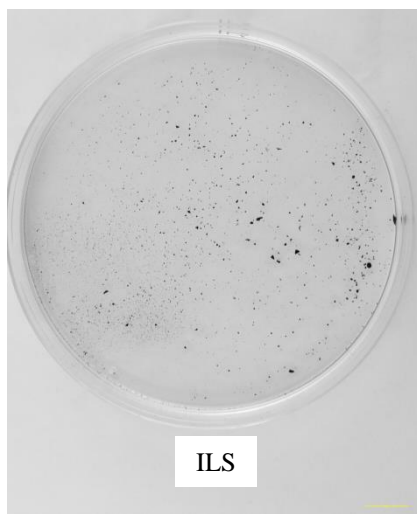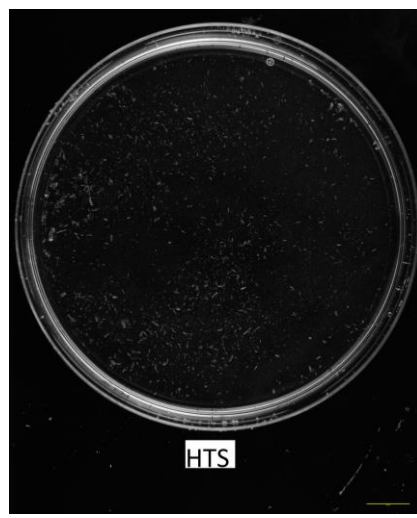

Supplement: Supplementary file 11 — Additional file 11: Figure S3. Particle sizes of untreated and pretreated lignocellulosic substrates. The figure contains images of the particle sizes of the untreated and pretreated straw substrates. The yellow scale bar in each image represents a length of 10 mm. [file 13068_2017_700_MOESM11_ESM.pdf]
